# A spatio-temporal agent-based approach for modeling the spread of zoonotic cutaneous leishmaniasis in northeast Iran

**DOI:** 10.1186/s13071-020-04447-x

**Published:** 2020-11-11

**Authors:** Mohammad Tabasi, Ali Asghar Alesheikh, Aioub Sofizadeh, Bahram Saeidian, Biswajeet Pradhan, Abdullah AlAmri

**Affiliations:** 1 Department of GIS, Faculty of Geodesy and Geomatics Engineering, K. N. Toosi University of Technology, Tehran, 19967 15433 Iran; 2 Infectious Diseases Research Center, Golestan University of Medical Sciences, Gorgan, Iran; 3 The Centre for Spatial Data Infrastructures and Land Administration (CSDILA), Department of Infrastructure Engineering, The University of Melbourne, Melbourne, VIC 3010 Australia; 4 The Centre for Advanced Modelling and Geospatial Information Systems (CAMGIS), Faculty of Engineering and IT, University of Technology Sydney, Sydney, Australia; 5 Department of Energy and Mineral Resources Engineering, Sejong University, Choongmu-gwan, 209, Neungdong-ro, Gwangin-gu, Seoul, 05006 Korea; 6 Earth Observation Center, Institute of Climate Change, Universiti Kebangsaan Malaysia, UKM, 43600 Bangi, Selangor Malaysia; 7 Department of Geology & Geophysics, College of Science, King Saud University, P.O. Box 2455, Riyadh, 11451 Saudi Arabia

**Keywords:** Zoonotic cutaneous leishmaniasis, Susceptible-Exposed-Infected-Recovered model, Geospatial information system, Agent-based model

## Abstract

**Background:**

Zoonotic cutaneous leishmaniasis (ZCL) is a neglected tropical disease worldwide, especially the Middle East. Although previous works attempt to model the ZCL spread using various environmental factors, the interactions between vectors (*Phlebotomus papatasi*), reservoir hosts, humans, and the environment can affect its spread. Considering all of these aspects is not a trivial task.

**Methods:**

An agent-based model (ABM) is a relatively new approach that provides a framework for analyzing the heterogeneity of the interactions, along with biological and environmental factors in such complex systems. The objective of this research is to design and develop an ABM that uses Geospatial Information System (GIS) capabilities, biological behaviors of vectors and reservoir hosts, and an improved Susceptible-Exposed-Infected-Recovered (SEIR) epidemic model to explore the spread of ZCL. Various scenarios were implemented to analyze the future ZCL spreads in different parts of Maraveh Tappeh County, in the northeast region of Golestan Province in northeastern Iran, with alternative socio-ecological conditions.

**Results:**

The results confirmed that the spread of the disease arises principally in the desert, low altitude areas, and riverside population centers. The outcomes also showed that the restricting movement of humans reduces the severity of the transmission. Moreover, the spread of ZCL has a particular temporal pattern, since the most prevalent cases occurred in the fall. The evaluation test also showed the similarity between the results and the reported spatiotemporal trends.

**Conclusions:**

This study demonstrates the capability and efficiency of ABM to model and predict the spread of ZCL. The results of the presented approach can be considered as a guide for public health management and controlling the vector population
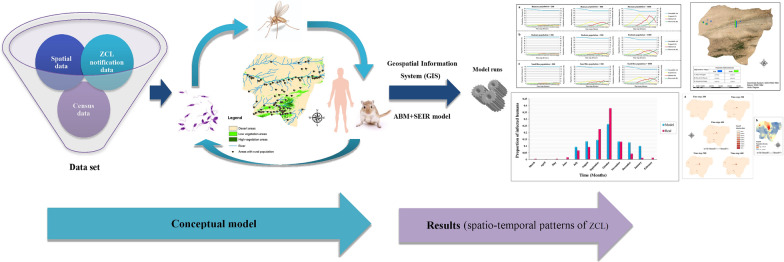
.

## Background

Two types of leishmaniases, visceral leishmaniasis (VL) and cutaneous leishmaniasis (CL), are common in Iran. The CL has two forms in Iran, zoonotic cutaneous leishmaniasis (ZCL) and anthroponotic cutaneous leishmaniasis (ACL). In the high-risk areas of CL in Iran, *Leishmania major* and *L. tropica* are the most common parasite agents causing ZCL and ACL, respectively [1].

Approximately 12 million humans are infected, and about 1.5 to 2 million new cases of CL occur each year worldwide [2]. More than two-thirds of new CL cases currently occur in six countries: Iran; Colombia; Algeria; Syria; Brazil; and Afghanistan [3]. Approximately 22,000 CL cases are reported in Iran each year, about 80% of which are ZCL [1, 4]. The ZCL occurs more often in rural areas and is endemic in Golestan Province located in northeastern Iran [5–7]. In 2015, 573 new cases were reported in this province, and the incidence of disease was 31.7 per 100,000 people [8]. According to research conducted in this province [5, 9], the primary and secondary reservoirs of the ZCL are two rodent species, namely *Rhombomys opimus* and *Meriones libycus*, respectively.

There are two transmission cycles for this disease: wild (zoonotic) cycle and domestic cycle. In the wild cycle, the sand fly of the *Phlebotomus caucasicus* group, including *Ph. mongolens*, *Ph. caucasicus*, and *Ph. andrejivi* as a vector, transmits the parasite from an infected wild rodent to a susceptible wild rodent (wild rodent-sand fly-wild rodent). But, in the domestic cycle, *Phlebotomus papatasi* as a vector, transmits the parasite (*Leishmania major*) from an infected human/wild rodent to a susceptible human/wild rodent [1, 4, 10].

According to Weber [11], environmental components, mainly location, are an important factor in the outbreak of diseases. Geospatial information systems (GIS) can be applied as a monitoring system to better track the route of infection in the propagation of diseases, which leads to the design of control strategies [12–14]. Several spatial analyses have been used to model leishmaniasis worldwide. Seid et al. [15] used statistical analysis and GIS to produce a risk map of CL based on spatial components in Ethiopia. In Iran, Mollalo et al. [16] stated that environmental factors significantly affect the spread of the ZCL. In their study, due to the environmental factors, the spatial spread of ZCL was limited to the northern and northeastern low-lying areas. Similarly, there are several studies on CL distribution in endemic regions, including deserts, plains, low-altitude, and high-population regions, which have a clustering pattern of the CL [17, 18]. Additionally, some CL studies have applied regression models [19, 20]. In India, Sudhakar et al. [21] generated predictive risk maps using GIS. Furthermore, many current studies have modeled the vectors and reservoirs of leishmaniasis in high-risk regions by GIS [22–24].

While the above studies are beneficial, researchers have also criticized them. For instance, Epstein [25] pointed out these models were inappropriate to present complex systems such as individual behavioral components and socio-ecological complex relations for disease modeling. In other words, these models ignore direct contact between humans, vectors, reservoirs, and the environment in disease modeling and mostly consider uniform mixing [26], which is not the case as individual agents interact with each other [27, 28]. Besides, they assumed humans as aggregate individuals, ignoring the heterogeneity of the population and key individual-based behaviors. Hence, heterogeneous individual agents operating over various social and geospatial spaces lead to explore the diverse view of disease dynamics [29].

Agent-based models (ABMs) are good alternatives to classical mathematical models [30]. Classical mathematical models account for a homogenous population and do not consider the dynamic interactions between entities (agents) within the complex system. These approaches also do not define the characteristics of the agents individually but in a group. In contrast, ABMs divide a synthetic population into different sections (types) and incorporate behaviors of agents within the system. In ABMs, known as a bottom-up approach, the behavior of the system is a result of collective interaction between individual agents. Therefore, considering heterogeneity in ABMs allow different agents in the same situations to make different actions [31]. This approach is more like to represent the actual condition and leads to a better understanding of the socio-ecological interactions than other methods [32].

Linking ABMs to spatial analysis leads to find and explore the complexity of disease spread over space [33]. Therefore, ABM has been applied to several epidemiological studies, including swine flu H1N1 [25], chikungunya [34], influenza [35], tuberculosis [36], malaria [33], cholera [37] and ZCL [38, 39]. Little consideration has been given in using ABM and GIS for ZCL. In a few research projects, having worked on this issue, researchers did not simulate the entire ZCL disease transmission cycle including not considering the rodents and their behaviors, not simulating all the main factors affecting ZCL spread such as humans and sand flies, and, or not modeling the individual-level reproduction behaviors of rodents. Also, in these studies, the temporal pattern of ZCL was not simulated. Based on the mentioned limitations, rodents and their behaviors in our study will also be simulated, and the temporal pattern of ZCL spread for one year will be explored and evaluated. Moreover, our proposed ABM explores the spread of ZCL with respect to the interactions between humans, vectors, reservoirs, and the environment in Maraveh Tappeh County, in the northeast region of Golestan Province in northeastern Iran, which is endemic for ZCL. In this study, GIS will also be employed to show the agent’s geospatial location and movement so that the model can indicate ZCL’s spatio-temporal diffusion through a set of interactions. Hence, the main aim of this work is to develop an ABM, which integrates GIS and an improved Susceptible-Exposed-Infected-Recovered (SEIR) model to analyze and evaluate the ZCL outbreak at a fine granularity (i.e. microscale) in an endemic area.

## Methods

This section includes the study area, data collection, modeling, and model verification and validation. In the study area section, the case study is described. The input data set, including ZCL notification data, spatial, and census data, are presented in the data collection section. The model is introduced in the modeling section, which consists of three parts, including a brief overview of the proposed model, design concepts, and details of the model. In the remainder of the methods section, model verification and validation are explained.

### Study area

**Fig. 1:**
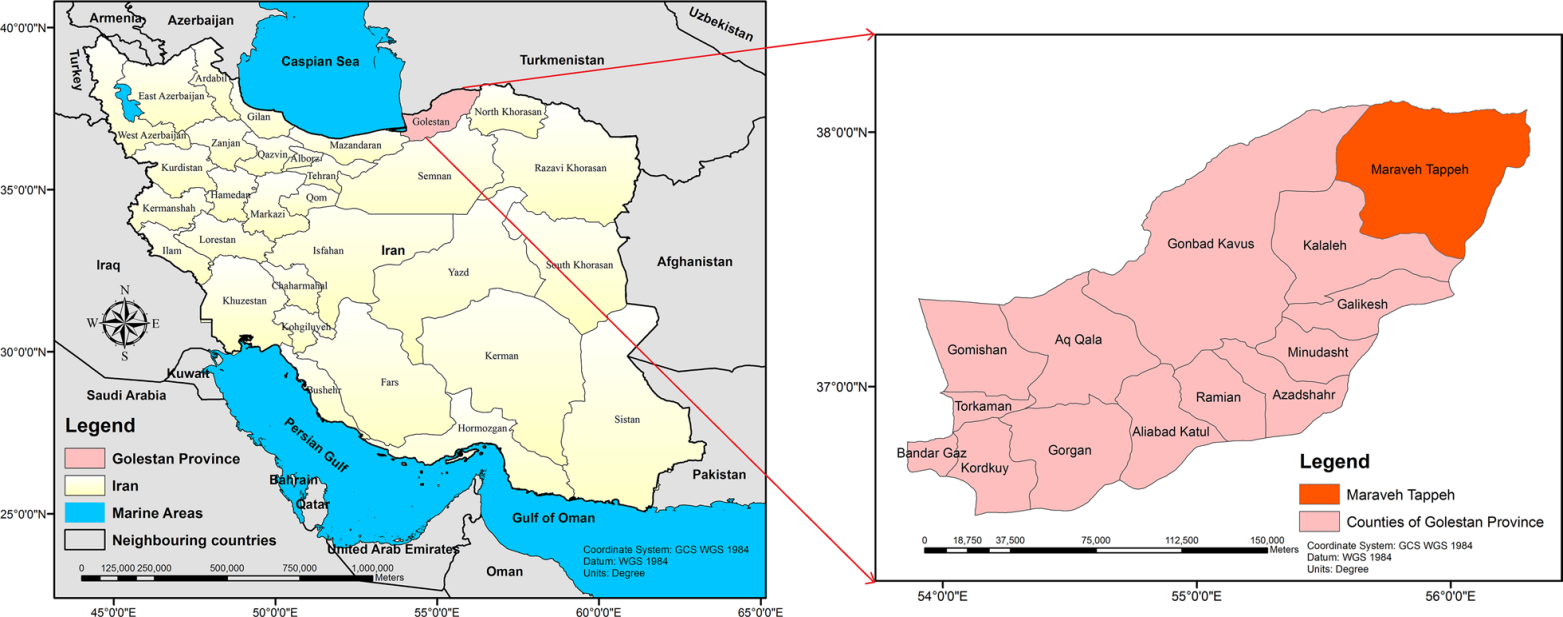
Location of the study area, Maraveh Tappeh, Golestan, Iran. The maps were illustrated using ArcMap version 10.2 based on geospatial data obtained from National Cartographic Center (NCC), Iran

Figure 1 shows the geographical location and the extent of the study area. Maraveh Tappeh County consists of 4 rural districts and 96 villages; its climate varies from the hot desert in the north to cold semi-humid in the south of the county, with warm summers, mild winters, and a great deal of sunshine throughout the year. The maximum temperature varies from 40.6 °C to 35.6 °C in summer. In the southern regions, predominantly in the mountains, land cover often is semi-forest and rocky soil. In contrast, the northern regions are mostly semi-desert and desert and are covered by soft soil, which represents favorable habitats for rodents, the reservoir hosts of ZCL. Suitable habitats of these rodents have large colonies where are composed of several subgroups and create networks of underground burrows. Most rodent activities can be seen during spring and summer (April to October), and these rodents become more visible during the night and in the early morning outside of their burrows. This activity decreases considerably when the temperature reduces in fall and winter. Having suitable and permanent weather in the underground burrows prepares rodent blood for phlebotomine sand flies in the study area. The favorable temperature for sand fly survival is 35–45 °C in summer within these burrows [7].

### Data collection

**Fig. 2:**
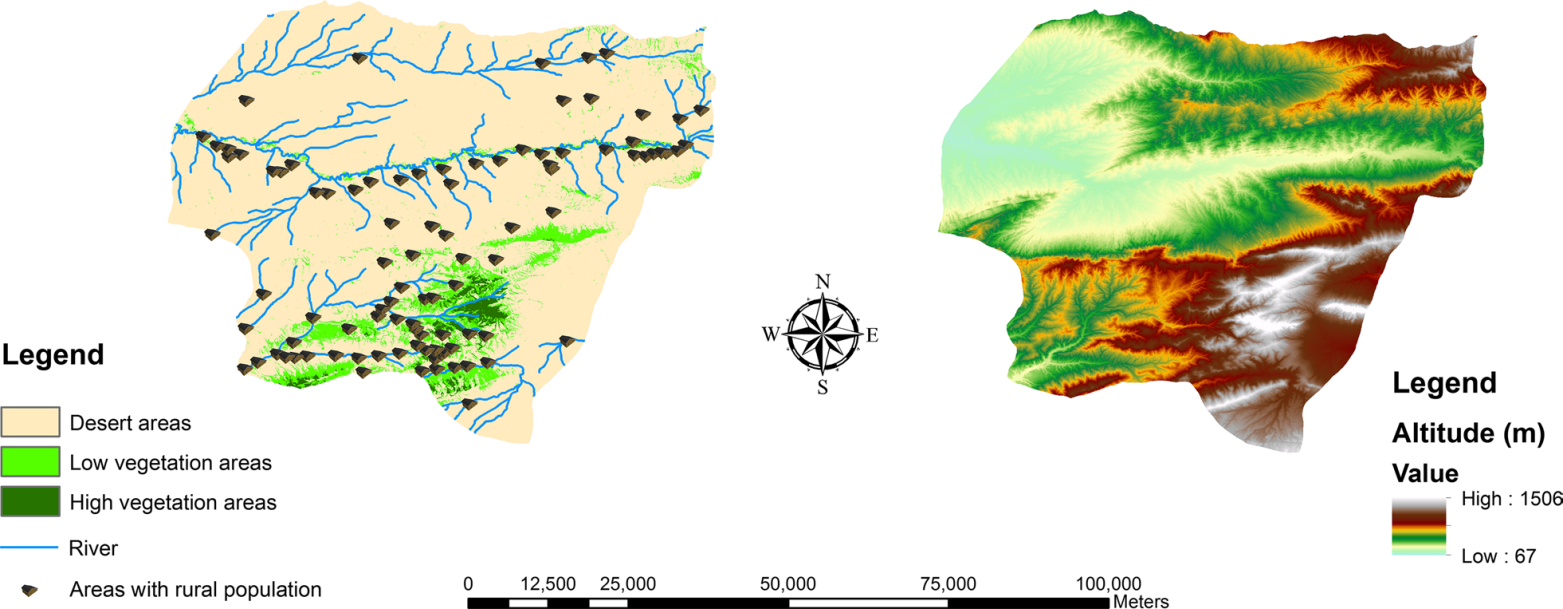
Input evidence maps for the ABM. Both normalized differentiated vegetation index (NDVI) and digital elevation model (DEM) were extracted from the United States Geological Survey (USGS) (https://gdex.cr.usgs.gov/gdex/) and were mapped in ArcMap version 10.2. These indexes were collected from the ASTER platform at 90 meters resolution

Based on the authors’ field observations and surveys and other leishmaniasis-related studies [16–18], the following parameters were chosen to be the main variables considered in the ABM generated by this study: rural populations, desert areas, altitude, and rivers (Fig. 2).

The input data set were generated from publicly available data sources as follows. The ZCL notification data were obtained from the Center for Disease Control and Prevention (CDC). The ZCL data only include people that went to health centers with clinical symptoms of ZCL, and the result of their microscopic test was positive. The landscape data were generated by the National Cartographic Center (NCC) and the United States Geological Survey (USGS). The data will be used in the county scale, and the field unit of the model is 100 × 100 m. Both NDVI and DEM were resized to the resolution of 100 m in ArcMap version 10.2 (ESRI, Redlands, CA, USA). In addition, the census data were collected from the records of national statistic organizations. It should be mentioned that all data (Table 1) were prepared in ArcGIS.

**Table 1:** Input data set

Data set	Type			Source
ZCL notification data	Monthly ZCL cases from 2011 to 2016 at the village level			Center for Disease Control and Prevention (CDC) of Golestan Province, Iran
Spatial data	Vector	County boundary, villages and rivers		National Cartographic Center (NCC), Iran
	Raster	Normalized Differentiated Vegetation Index (NDVI)	Desert areas	United States Geological Survey (USGS), downloaded from its website (https://libra.developmentseed.org/)
			Low vegetation areas	
			High vegetation areas	
		Digital Elevation Model (DEM)	Elevation map	United States Geological Survey (USGS), downloaded from its website (https://gdex.cr.usgs.gov/gdex/)
Census data	Villages population			Statistical Center of Iran, downloaded from its website (https://www.amar.org.ir/english/)

### Modeling

#### Overview

Our model is an agent-based framework that simulates human, sand fly, rodent, and environment behaviors regarding the spread of ZCL. Designing a spatial model of the ZCL spread as a complex geospatial system is the main objective of the model, including spatial and non-spatial data and agent objects. Hence, our approach considers the spatial heterogeneity and spatial behaviors explicitly. The agent-based simulation platform of NetLogo version 6.1.1 (Northwestern University, Evanston, IL, USA) was used for modeling. The model is described in Additional file 1. ZCL transmission is a complex process, which depends on many parameters. To skip these complexities and to prevent the deviation of the model from its original purpose, some simplifying assumptions are described in the following sections.

**Fig. 3:**
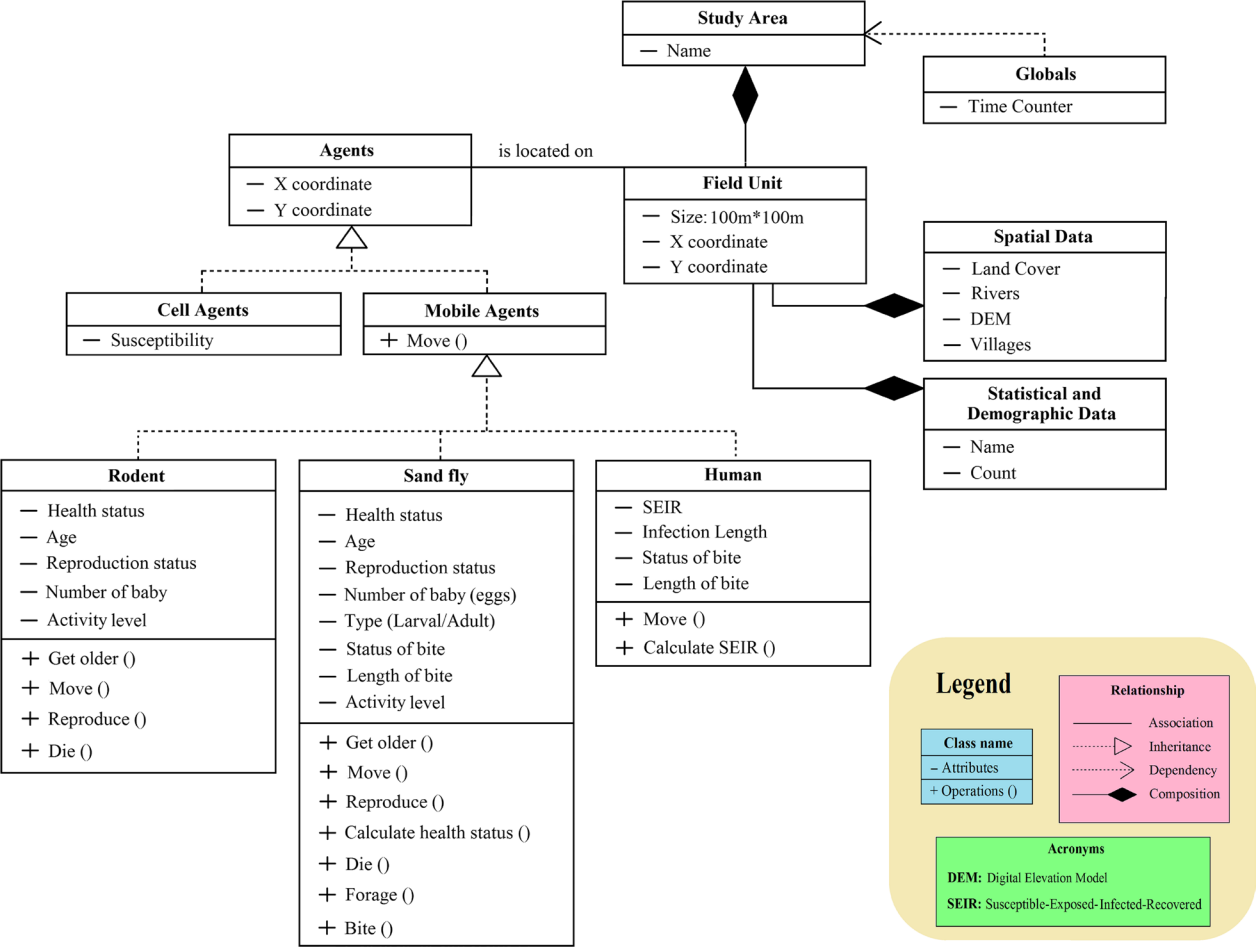
UML class diagram of the ZCL model

We considered various aspects of known sand fly and rodent behaviors and several ZCL characteristics. Simulation results provide some initial insight into how certain sand fly, rodent, and human characteristics might change the severity of the ZCL spread. To understand the dynamics of the ZCL outbreak and the spatially behavioral heterogeneity, various features are considered for agents (sand fly, rodent, human, and environment). The unified modeling language (UML) diagram applied in this research is shown in Fig. 3. As seen in this figure, mobile agents and cell agents are two kinds of agents considered in the model. Mobile agents are separated into three classes: sand fly, rodent, and human. Each of them has its own set of variables and counters, including Boolean variables for each infection state. Generally, ZCL is transmitted between mobile agents in four ways (human-sand fly-human, human-sand fly-rodent, rodent-sand fly-rodent, and rodent-sand fly-human) by infected female sand flies [2]. Foraging and biting human/rodent are two main behaviors of the sand fly agents. Sand flies and rodents have variables for their age, health status (such as susceptible and infected), reproduction, and environment (known as a “patch”) on which they will be initialized. At any point in time, human agents may reside in one of four states: susceptible; exposed; infectious; or recovered. In addition, cells define the ABM environment in which mobile agents are moving, interacting, and doing their tasks. Each of these cells is a statistic agent. They generate the risk value (susceptibility) of their areas based on the number of human infections occurred in each place, which is calculated according to observations of the cell agents. Furthermore, we assumed that environmental factors in each field unit are constant. They show the habitat suitability, which can affect the spread of ZCL.

**Fig. 4:**
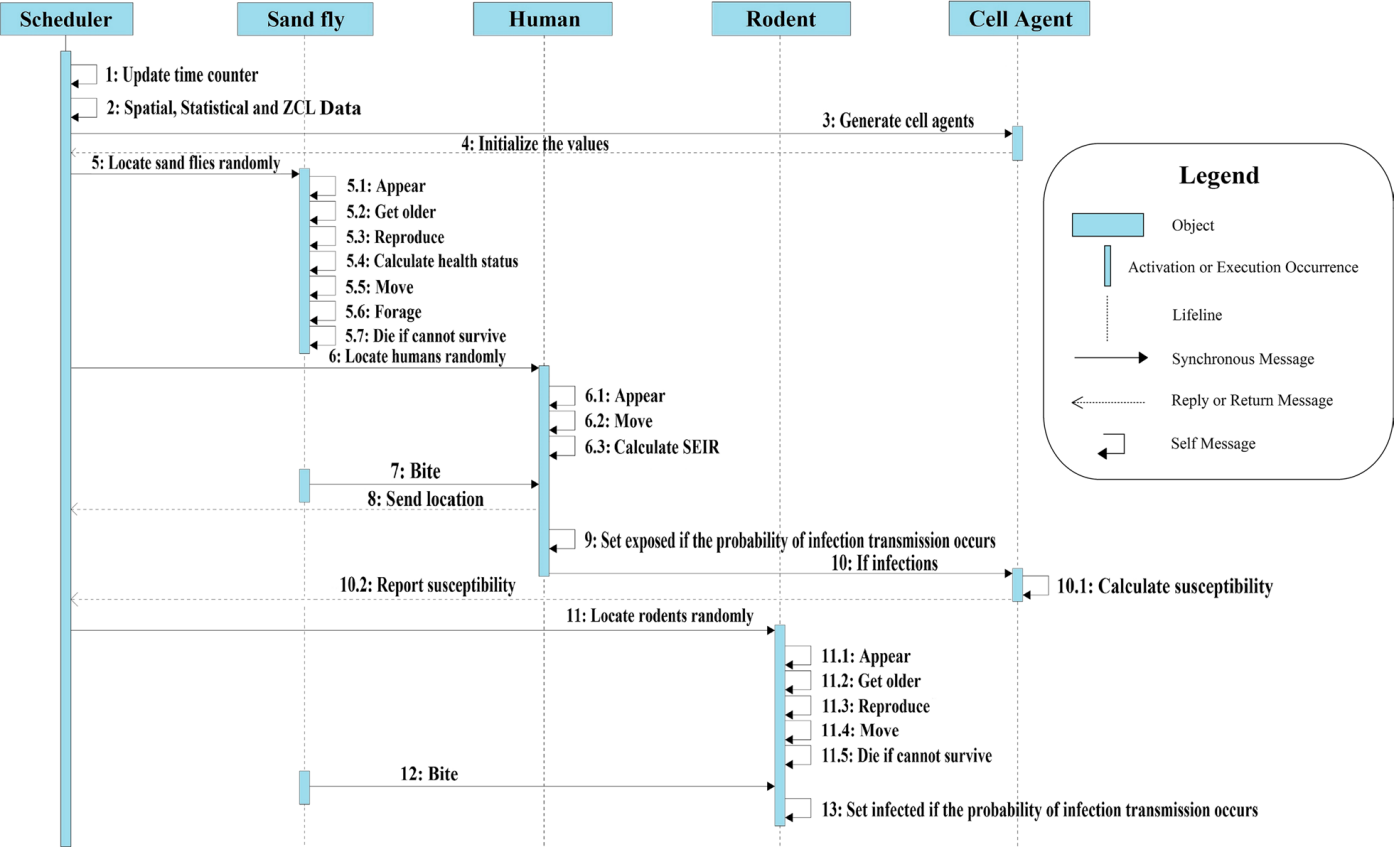
Display of the ZCL model by utilizing the UML sequence diagram

To create a model as simple as possible while preserving the required complexity, we assumed that the time steps are short enough to gain better dynamics and long enough to prevent dispensable overhead. After defining the time steps, we can set the other parameter values. The disease parameters and the number of contacts among agents depend on the time steps; thus, they should correlate to the timing of the model. The model is updated each 8 h intervals, called ticks in this manuscript. Figure 4 presents a UML sequence diagram that is an overview of the entire scheduling process. In this diagram, we can see an outline of the sequence of procedures and the schedule of interactions between various agents at each time step. Each procedure is specified according to a specific class of agents based on the UML class diagram.

First, the time counter is updated at each discrete time step. Then, the field unit values are initialized based on the spatial, statistical, and ZCL data. Next, cell agents are placed in the environment. Then, sand flies appear in the environment at various random locations. Sand flies move to low altitude areas and begin to forage and bite humans or rodents if the time is between 00:00 h and 8:00 h on any given day of spring or summer, and they have completed the larval stage. Then, humans appear in their village centers and move freely through the environment. Next, rodents appear in the environment with random positions and move to the desert areas where their favorite habitats are. Agents of the same type are processed at each time step by utilizing a random sequence. Some procedures are only operated under specific circumstances, e.g. the recover function only begins after a human is infected.

#### Design concepts

To create the model, we utilized the knowledge from previous models of infectious diseases. The ZCL progresses like an improved SEIR model where a human status can be changed in different stages depending on whether and when an individual contracts the disease. In addition to the progression of the ZCL, the spread of this disease primarily depends on interactions between sand fly, human, and rodent. Other factors play a role in disease spread, including the spatial and statistical data of the case study, affecting the spatio-temporal dynamics of ZCL.

Disease spread is emergent behavior captured in our model and is governed by the behaviors and characteristics of the mobile agents. Although agent behaviors and decisions (which different agent types need to make. For instance, in each iteration, sand fly agents decide whether they want to move or not) are strictly defined, stochasticity is present in each. Sand flies are capable of sensing other mobile agents (human/rodent) and cell agents (low altitude areas/rivers). Humans can sense all patch variables, including patch color and population density. When cell agents perceive the interactions among sand fly, human, and rodent through their landscape, ZCL susceptibility is updated. In the interactions of the model, we can explore the complex trajectories of ZCL outbreaks by disorganized interactions among agents and between agents and the environment at a microscale. Sand flies interact with other mobile agents (human/rodent) during the biting and foraging procedure. Also, when sand flies arrive at the river patches, they lay eggs. Moreover, when a human is bitten by an infected sand fly, the risk value of the field units where a human is located increases. Many processes in the model involve some levels of stochasticity. We randomly assigned some characteristics of the agents (e.g. the age of sand fly, rodent’s reproduction status, and initial location of mobile agents) at the initialization of the model. Biting and disease transmission are both stochastic procedures controlled by a user-controlled slider. The mobile agents are collectives of agents that have different locations, movement patterns, and variables. At certain times of day (00:00–08:00 h), the sand fly moves and starts to bite and forage. When a sand fly reaches adulthood, it moves and lays eggs for the entirety of the lifespan. By observing the model, the user can track the statistics such as the number of humans with various statuses (according to the SEIR model) per iteration and the susceptibility of each cell. The user also shows the highest infection sites in the study area and the temporal pattern of the ZCL in one year of simulation.

#### Details

The initialization of the model depends on the landscape data of the study area, which includes the spatial and non-spatial data. We determined the parameter values according to related works, some of the most authoritative websites, and experts’ judgments. All parameters and their values are described in Table 2.

**Table 2:** Summary of input parameters and their values used in the ABM model

Parameter	Description	Values	Source
Sand fly	Flight range	500 m	[40]
	Active period	Every day from 00:00 h to 8:00 h in spring and summer	
	Time interval between meal (forage/bite) and reproduce	5 days	
	Lifespan	80 days	
	No. of days a sand fly (egg) reaches the end of the larval stage and turns into the adult sand fly	30 days	
	Max no. of eggs produced by a sand fly in its whole life (lifespan)	100	
	Max no. of eggs produced by a sand fly in each discrete time step (reproduction)	30	
	Carrying capacity (maximum number of existing sand flies in each time step)	1000	Experts’ judgments
	Sensory range for move	500 m	
	Sensory range for meal (bite/forage)	300 m	
	No. of days a sand fly spends in exposed state (E_S_)	10 days	[41]
	Baseline DMR (for larvae and adults) ($\rho$)	0.1	[33]
	Exponential mortality increases with age ($\sigma$)	0.04	
	Degree of mortality deceleration ($\varepsilon$)	0.1	
Rodent	Movement range	300 m	Experts’ judgments
	Active period	Every day from 00:00 h to 8:00 h in spring and summer	[42]
	Lifespan	3 years	
	No. of days a newborn rodent turns into the adult rodent	100 days	
	Max no. of rodent reproductions during their lifespan	50	
	Max no. of rodent reproductions per reproduction	5	
	Carrying capacity (max. no. of existing rodents in each time step)	1000	Experts’ judgments
	Sensory range for move	200 m	
Human	Movement range	50 m	
	Active period	Every day from 8:00 h to 24:00 h	
	Initial no.	100–2000	User settable
	No. of days a human spends in the exposed state (E_H_)	120 days	[41]
	No. of days a human spends in the infected state (I_H_)	150 days	
	The probability of transiting from recovered state to susceptible state (γ)	0.02	[43]
Infection transmission cycle	Transmission probability of infection from sand fly to human and rodent (α)	0.6	[34]
	Transmission probability of infection from human and rodent to sand fly (β)	0.275	

Based on Fig. 3, mobile agents have particular coordinates, their state changes under different circumstances, and they are enabled to move. The states of cell and mobile agents can change over time. Even though both kinds of agents are placed in a specified location within the study area, only the positions of mobile agents can vary at each time step. Mobile agents can move and explore the landscape based on their behaviors and determined rules. In addition, cell agents are static and do not move. Below, we list and describe the function of each sub-model.

Although collecting the distribution frequencies of vectors and reservoirs can be a useful tool for more accurate ZCL modeling, it is costly and time-consuming and needs special skills [8, 22]. Therefore, we assume that sand flies randomly appear in the environment. If age is 30 days (this means that as sand flies emerge into the adult stage and the larval stage ends [40]) and if time is between 00:00 h and 8:00 h in spring and summer [40], they start to search in their Moore neighborhood (is defined on a two-dimensional square lattice and is composed of a central cell and the eight cells that surround it) where is within a circle with a certain radius (sensory range for move). Inside this area, they move toward their favorite site where the altitude is minimum. In addition, the maximum distance of the sand fly movement at each time step is 500 m. When adult sand flies are arriving at the end of their active period each day, they rest and do not move.

Sand flies can sense humans and rodents within their perception range (sensory range for a meal). Therefore, if an infected female sand fly is within a distance of this range, the sand fly will attempt to bite humans and rodents. The chance that the bite will be successful is user-defined through the transmission probability of infection from sand fly to human and rodent [34]. If the bite is successful, and if humans and rodents were susceptible, the humans’ status becomes exposed, and rodents become infected until the end of their lifespan [42]. Sand flies need blood for the development of eggs in their reproductive system [44]. Hence, if rodents and humans move to the perception range of sand flies, and if all of them (sand fly, rodent, and human) are infected or susceptible, then sand flies forage humans and rodents to reproduce. Moreover, if infected humans and rodents are within the perception range of susceptible sand flies, according to a certain probability (β) [34], sand flies enter the exposed state. Once the exposed period has passed, sand flies move from exposure to infectious and remain infectious for the remainder of their lifespan [40]. Consequently, infected sand flies are ready to infect other susceptible mobile agents.

Unlike other traditional CL transmission models, we consider senescence (biological aging) of sand flies. The ABM defines age-specific mortality rates for adult sand flies and the larvae (i.e. the probability of death for sand fly agents increases with their age). According to Styer et al. [45], we used the logistic mortality model as follows:

1 $$DMR_{(Age)} = \frac{{\rho \times e^{Age \times \sigma } }}{{1 + \frac{\rho \times \varepsilon }{\sigma } \left( {e^{Age \times \sigma } - 1} \right)}}$$

where $\rho$ is the baseline DMR (daily mortality rate), $\sigma$ is the senescent/aging component (the exponential mortality increase with age), $\varepsilon$ is the degree of mortality deceleration, and Age is the age of the sand flies. These coefficients and their values were presented in Table 2.

Since each time-step was set to eight hours in the model, we calculated the 8 hourly mortality rate (EMR) for each age of the sand flies (Eq. 2). In this regard, at every age, sand flies have a certain chance of dying, and this chance increases exponentially with age.

2 $$EMR_{(Age)} = \, 1 \, {-} \, \left( {1 - \, DMR_{(Age)} } \right)^{1/3}$$

Since rodent colonies, as well as riversides, are suitable areas for laying eggs [46], we set the following rules for the reproductive behavior of sand flies. Adult female sand flies reproduce under certain conditions, including passing at least the 5 days of their meal (forage and bite), the time is spring and summer, the number of existing sand flies in the model is less than the carrying their capacity, the number of their reproductions is less than the maximum number of their reproductions during sand fly’s lifespan, and if they locate at a 500 m radius of rodents and in the riverside. If all the above conditions are met, sand flies will produce a maximum of 30 new sand flies (eggs) randomly. If the parent sand fly is susceptible, exposed, or infected, a new sand fly will be susceptible too. New sand flies begin at age 0 and will reside in their location until their age reaches 30 days; at that time, they will move [40].

As the movement of sand flies affects the spatial movement of infection, the displacement of rodent agents leads to the spread of ZCL through the landscape. Accordingly, rodents appear in the study area with a random location. If time is between 00:00 h and 8:00 h on any given day of spring or summer, they move to their favorite places; desert areas are in their Moore neighborhood (sensory range for move), and they rest and do not move for the remainder of a day. Based on experts’ judgments, we have considered that the maximum distance of the rodent’s movement at each time step is 300 m.

The proposed model also implements a mortality process for rodents. The rodents die based on a specified number of time steps. The probability of death for a rodent increases with their age. In this regard, at every age, rodents have a certain chance of dying, which increases as the age of a rodent increases. These procedures were chosen to limit the total of rodent’s population.

Adult female rodents reproduce under certain conditions that include; time must be spring or summer, the number of existing rodents in the model is less than the carrying capacity, and the number of their reproductions is less than the maximum number of their reproductions during rodent lifespan. When all the above conditions are confirmed, rodents will produce a maximum of 5 new rodents randomly [42].

As in the real world, a human agent might or might not become infected by the disease. At the beginning of the simulation, humans appear in village centers, and they can move freely in their environment. A maximum distance of movement at each time step is assumed as 50 m. The whole population of the studied area cannot be simulated because of the computational burden. Hence, we have assumed that the number of humans initialized in the villages is determined by the population distribution in the census data. Moreover, because of the short simulation time of 1 year, no getting older, no dying, and no birthing occur for human agents. In other words, no humans enter or leave the model.

**Fig. 5:**
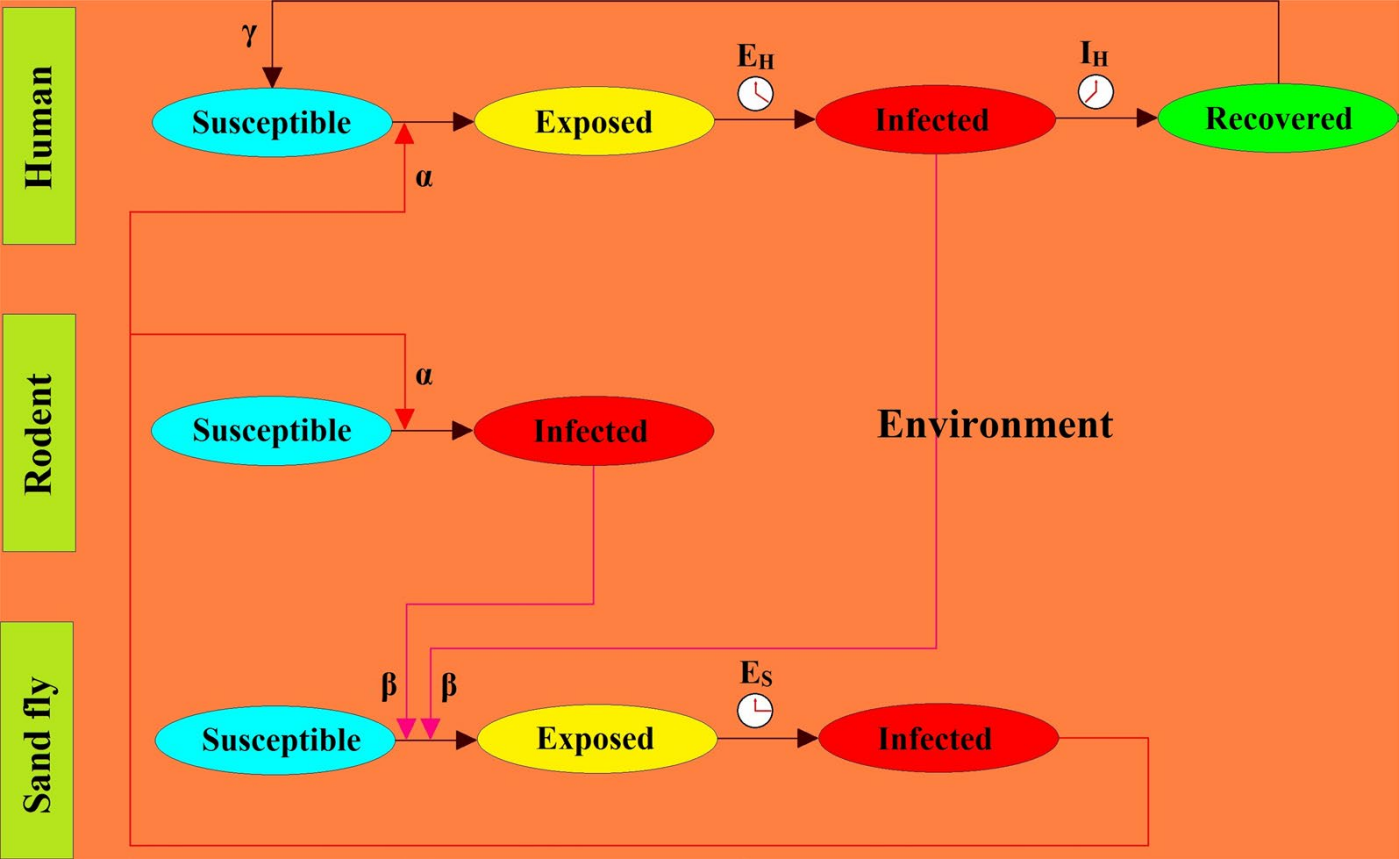
ZCL transmission through the interaction of sand fly, human, rodent, and the environment

Considering that many vector-borne diseases are known to have significant incubation periods (malaria, dengue, chikungunya, etc.), the SEIR model was chosen as its exposed state addresses the incubation period (E_H_) (Fig. 5). For humans, the susceptible state is when a human is vulnerable to infection by the pathogen. A human is exposed when he/she is carrying the infection but is not yet contagious. The infected state is when a human has started showing symptoms and is ready to infect a sand fly. Over time steps, all infected humans directly enter the recovered state after passing the infection period (I_H_). In addition, the recovered human can turn into a susceptible human with a certain probability (γ). Infection in sand flies follows a similar cycle, except they do not recover once they have been infected. We also assumed that the rodents only have two steps of the SEIR model: the susceptible state and the infected state. In other words, when an infected female sand fly bites a susceptible rodent, it immediately turns into the infected rodent with a non-zero probability (α), and it remains infectious for the remainder of the lifespan. When a susceptible female sand fly bites an infected human/rodent, it gets infected with a non-zero probability (β). If a sand fly acquires the pathogen, it multiplies in the sand fly until it reaches enough strength to infect some other susceptible human/rodent, thereby completing a cycle. This period is called the exposed period for sand fly (E_S_). Fig. 5 presents the structure of the SEIR model encompassing various components considered in the model.

Once a susceptible human is bitten by an infected sand fly, the value of cell susceptibility where the human is located will increase by one unit. Therefore, the susceptibility map is updated by changing the cell agents. Accordingly, locations with more susceptibility represent the high-risk cells where a human is infected by a sand fly. In the remainder of this work, this map will be applied to trace the spatial spread of ZCL over time steps.

### Model verification and validation

Before interpreting the results, it is essential to discuss the validation process. By model validation, we determine that the programming implementation of the abstract and conceptual model operates correctly [47]. We verify our model by conducting code walk-throughs, debugging software, looking for an incorrect implementation of conceptual models, and verifying the calculations to assure the model has no logical errors in the translation of the model into code. Performing these tests leads to matching the model with its intended design. To confirm that our proposed conceptual model is a reasonably correct representation of the real world, and to increase the credibility of the model, validation processes were executed.

To investigate the model, we ran four experiments varying key parameters of interest. In the first experiment, we tested three levels of the population size of mobile agents. In the second experiment, we investigated the impact of the infectious period of humans on the epidemic. The third experiment tested three levels of initially infected sand fly and rodent. In the fourth experiment, we explored how restricting movement of human effects on the ZCL spread. These experiments were conducted to gain more understanding of the dynamics of the ZCL outbreak under various conditions. All these experiments not only allowed the model performance evaluation and led to testing the inner validity of the model, but also demonstrated how our model could be applied to explore endemic ZCL. The model was implemented based on the parameters mentioned in Table 2. It should be noted that the results of the proposed model were extracted from the average of twenty runs of the model. To validate the model, we compared the simulation outcomes with ZCL notification data (see Table 1). For this purpose, the spatial and temporal pattern of ZCL is validated.

## Results

### Sensitivity analysis

#### Population size

**Fig. 6:**
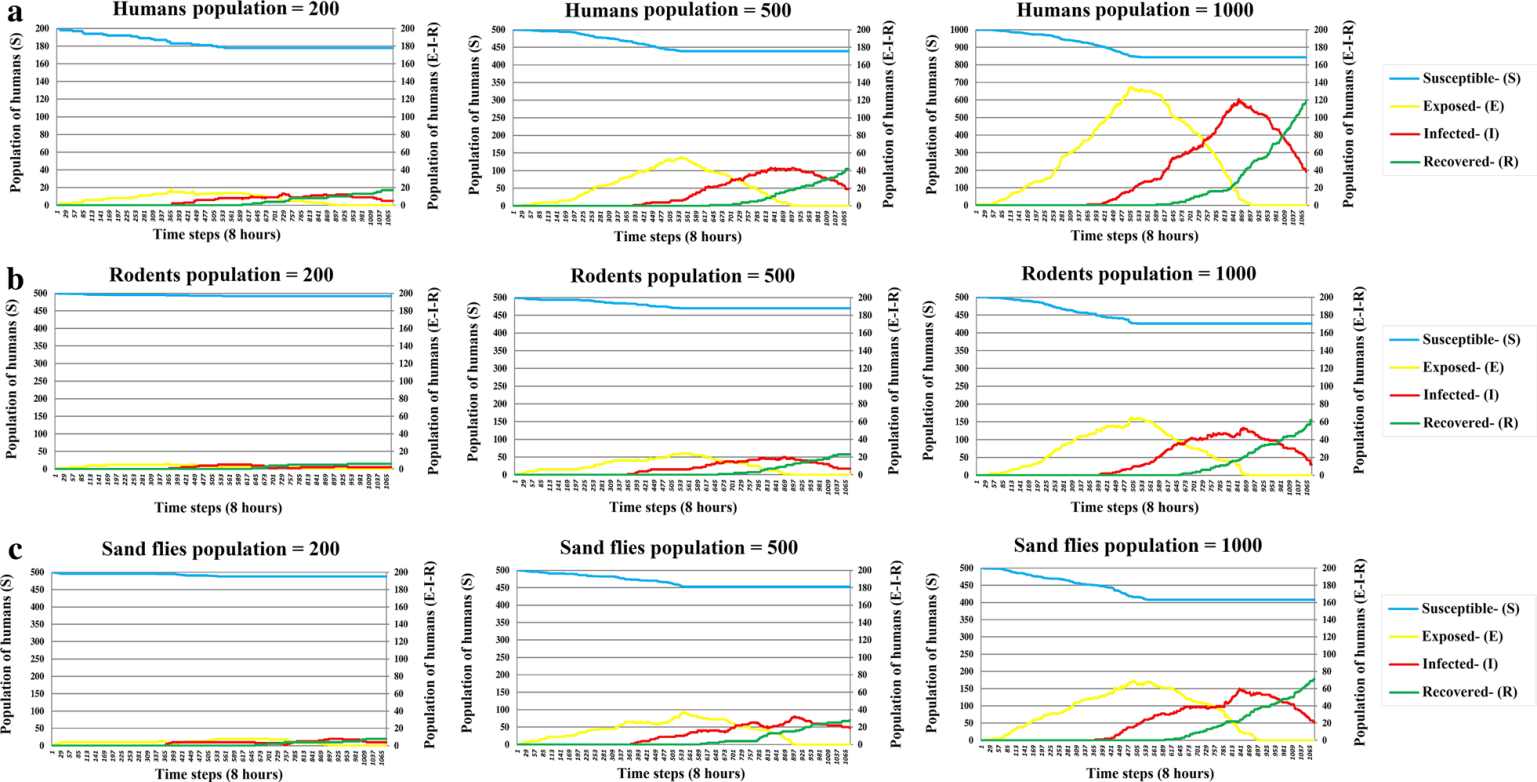
SEIR results with different initial populations (human (**a**), rodent (**b**), and sand fly (**c**)). Blue, yellow, red, and green lines refer to susceptible, exposed, infected, and recovered humans, respectively. The left vertical axis in all charts indicates the number of susceptible humans. The right vertical axis in all charts shows the number of exposed, infected, and recovered humans

Here we explore how a change to the population size of each mobile agent affects the ZCL outbreak. Figure 6a-c illustrates different statuses of human agents in the SEIR model within Maraveh Tappeh with various population densities of humans, sand flies, and rodents, respectively. This experiment tests how sensitive our model is to population sizes.

#### Infectious period of a human

**Fig. 7:**
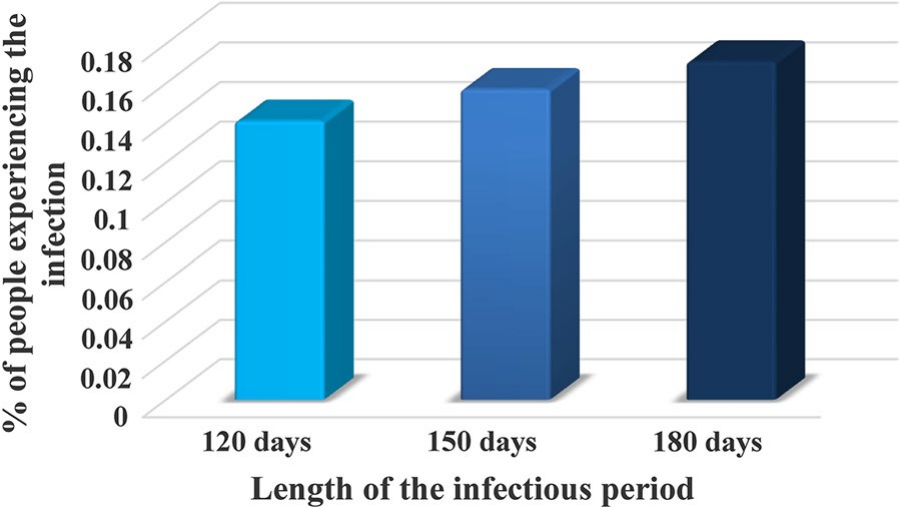
The human infection percentage throughout the epidemic in response to different infectious periods. The vertical axis of the chart indicates the percentage of infected humans. The horizontal axis of the chart refers to the infectious periods of humans

This parameter defines the duration of a period in which a human can infect susceptible sand flies. Figure 7 shows the impact of the duration of an infectious period by the different length from 120 to 180 days. Since the treatment process for infectious humans was not accounted in this model due to the simplification, it is assumed that all infected humans recovered after passing the infection period (I_H_) over time steps. The previous studies also indicated that the infection heals spontaneously [48, 49]. In Iran, the average period of I_H_ (without any treatments) is 150 days [41]. Therefore, these periods were considered for the model. A longer infectious period leads to a greater proportion of infections during the period of an epidemic.

#### Initially infected rodent and sand fly

**Fig. 8:**
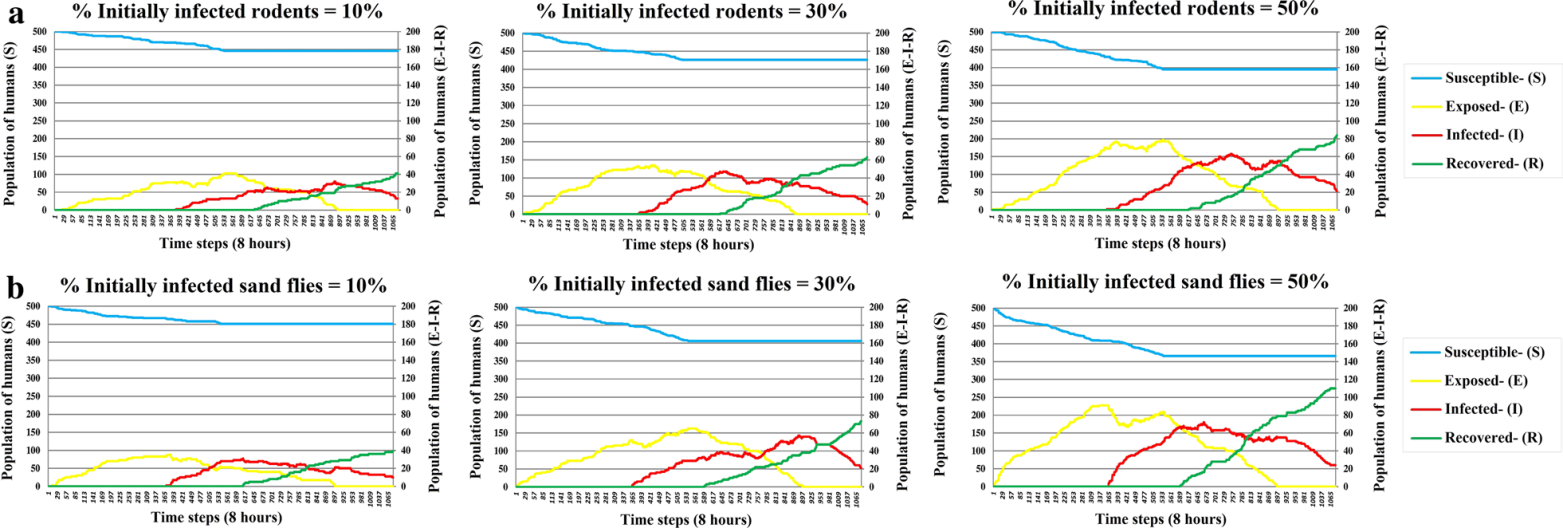
SEIR results with different initial infected populations (rodent (**a**) and sand fly (**b**)). Blue, yellow, red, and green lines refer to susceptible, exposed, infected, and recovered humans, respectively. The left vertical axis in all charts indicates the number of susceptible humans. The right vertical axis in all charts shows the number of exposed, infected, and recovered humans

Figure 8a, b shows that as the percentage of initially infected rodents and sand flies increases, the number of infected humans grows. This is not surprising because the percentage of initially infected rodents and sand flies is expected to proportionally affect the probability that a susceptible human will experience an infection. By comparing Fig. 8a, b, it can be stated that an infected sand fly plays a more effective role in disease spread relative to an infected rodent.

#### Restricting human movement

**Fig. 9:**
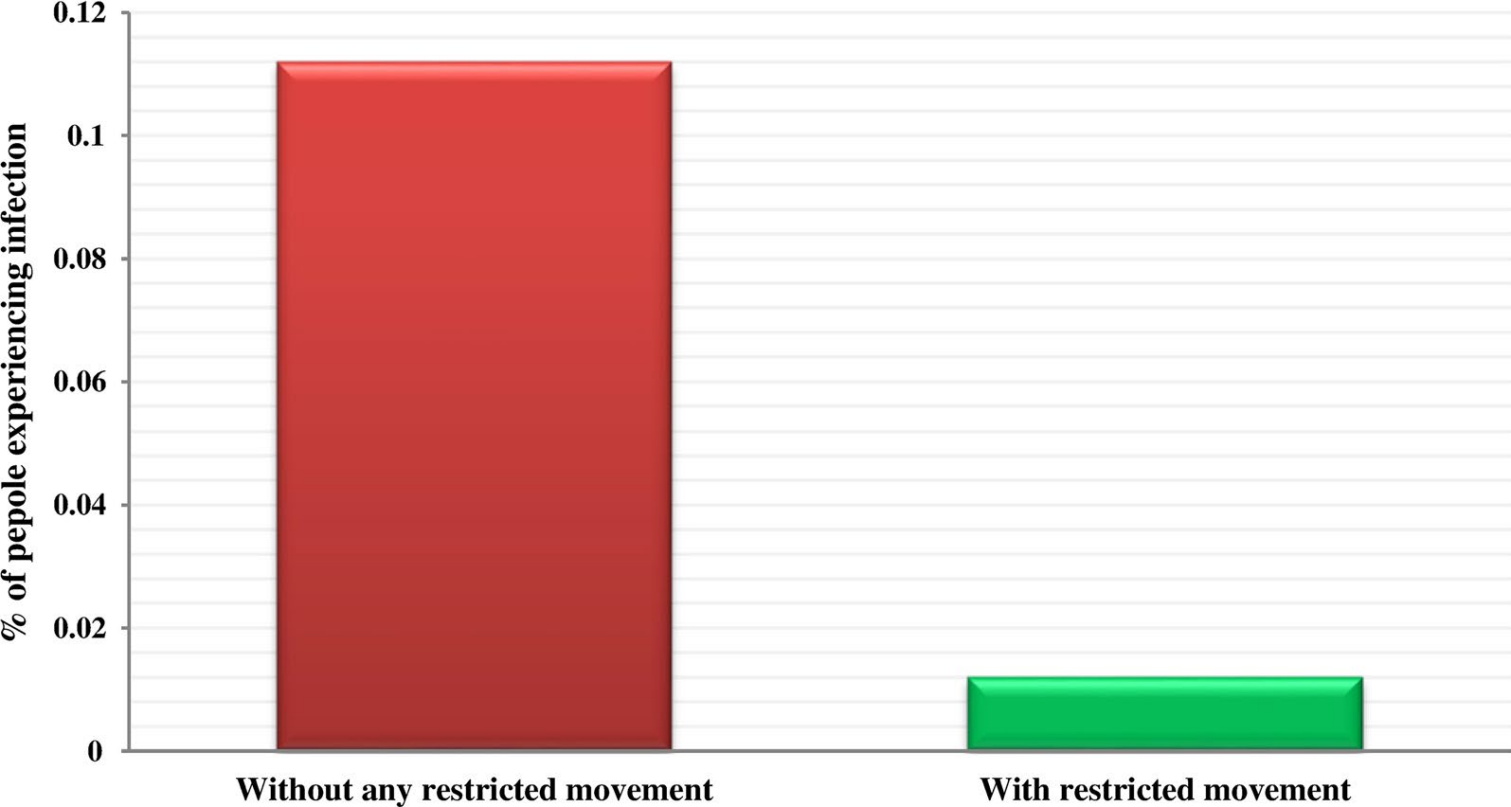
The human infection proportion in the case study in response to the two-movement rules. The red box indicates the percentage of infected humans for whom there was no movement restriction. The green box shows the percentage of infected humans for whom there was movement restriction

One way to prevent and control the ZCL is to shorten human appearances in the habitats of vectors and reservoir hosts (desert and low altitude areas, and rivers). In the first state, humans can move randomly in the environment. In the second state, humans only can move to the extent of their villages (restricted movement). To compare these two scenarios, all other parameters remain constant. Figure 9 shows the proportion of infected humans in response to these two scenarios. As expected, without any movement restrictions, the infections have been considerably increased while restricting movements can result in an approximately 10% reduction in the percentage of infected humans.

### Model validation

#### Spatial pattern

**Fig. 10:**
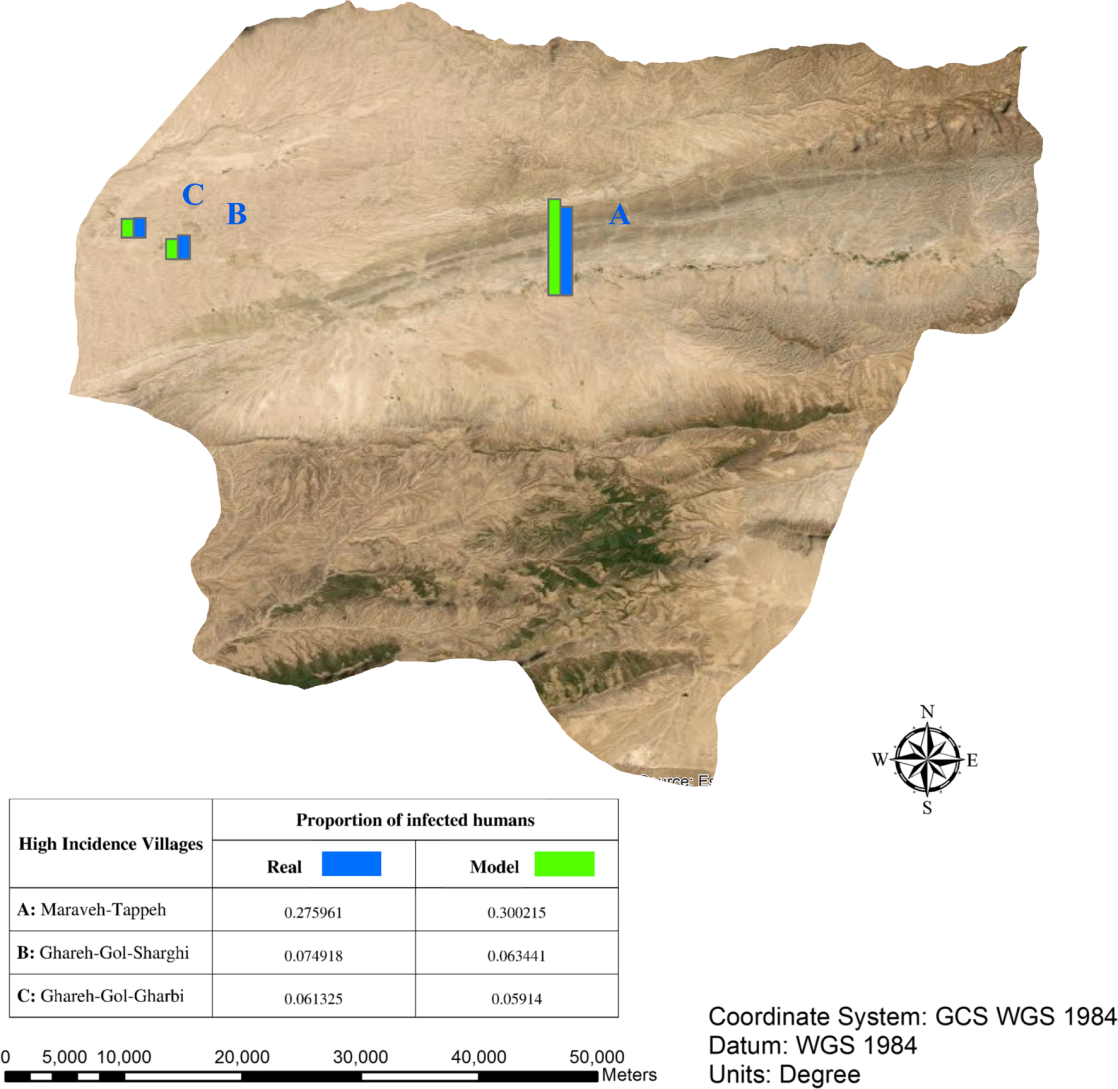
Comparison of simulation results with reality in terms of the proportion of infected humans in three endemic areas in the case study. This map was obtained from the United States Geological Survey (USGS) (https://gdex.cr.usgs.gov/gdex/) and was displayed in ArcMap version 10.2

**Fig. 11:**
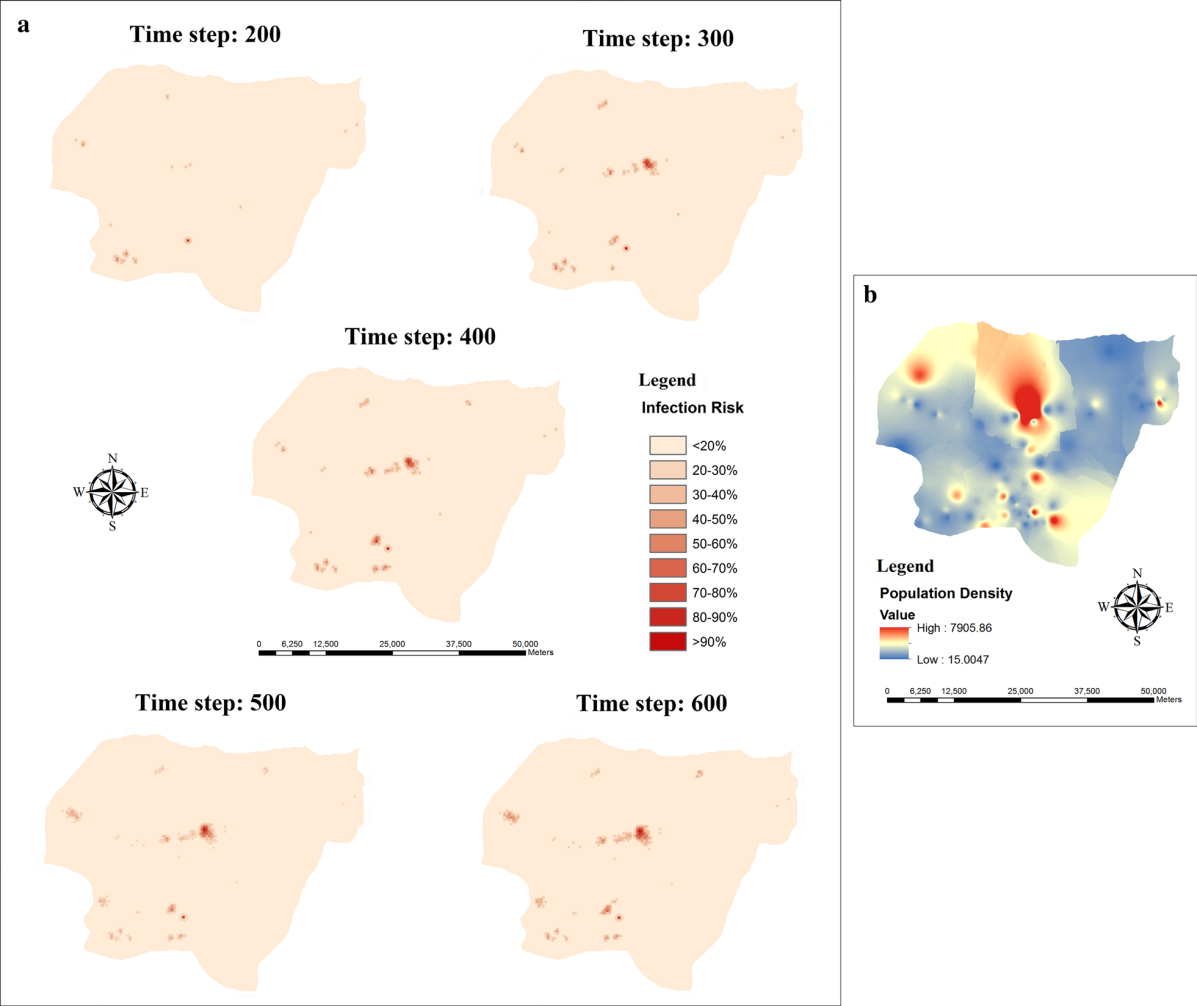
**a** Spatial pattern of ZCL during the consecutive time steps in the Maraveh Tappeh County. **b** Population density of village residents in the study area. In panel **a**, bold red areas indicate regions with the highest levels of the infection. In panel **b**, bold red areas show regions with the highest population density, and blue areas indicate the lowest population density. The maps were prepared by ArcMap version 10.2

Based on the information obtained from ZCL data, three villages in the case study are highly infected. They are Ghareh-Gol-Gharbi, Ghareh-Gol-Sharghi, and Maraveh-Tappeh. It could be because more desert and low altitude areas are located around these villages. It makes these villages more exposed to ZCL from sand flies. To evaluate the spatial pattern of ZCL, we compare the proportion of infected humans resulted from the simulation with ZCL data in these villages, as shown in Fig. 10. It is clearly observable that simulated results are almost consistent with real data. Furthermore, to track the infection path, we illustrate the spatial pattern of ZCL during the consecutive time steps, as shown in Fig. 11. High-risk areas show a high probability of ZCL exposure and infection. Figures 2 and 11 show that the spatial pattern of ZCL is much more localized around regions with high population density, desert, and low altitude areas, and starts to spread along rivers.

#### Temporal pattern

**Fig. 12:**
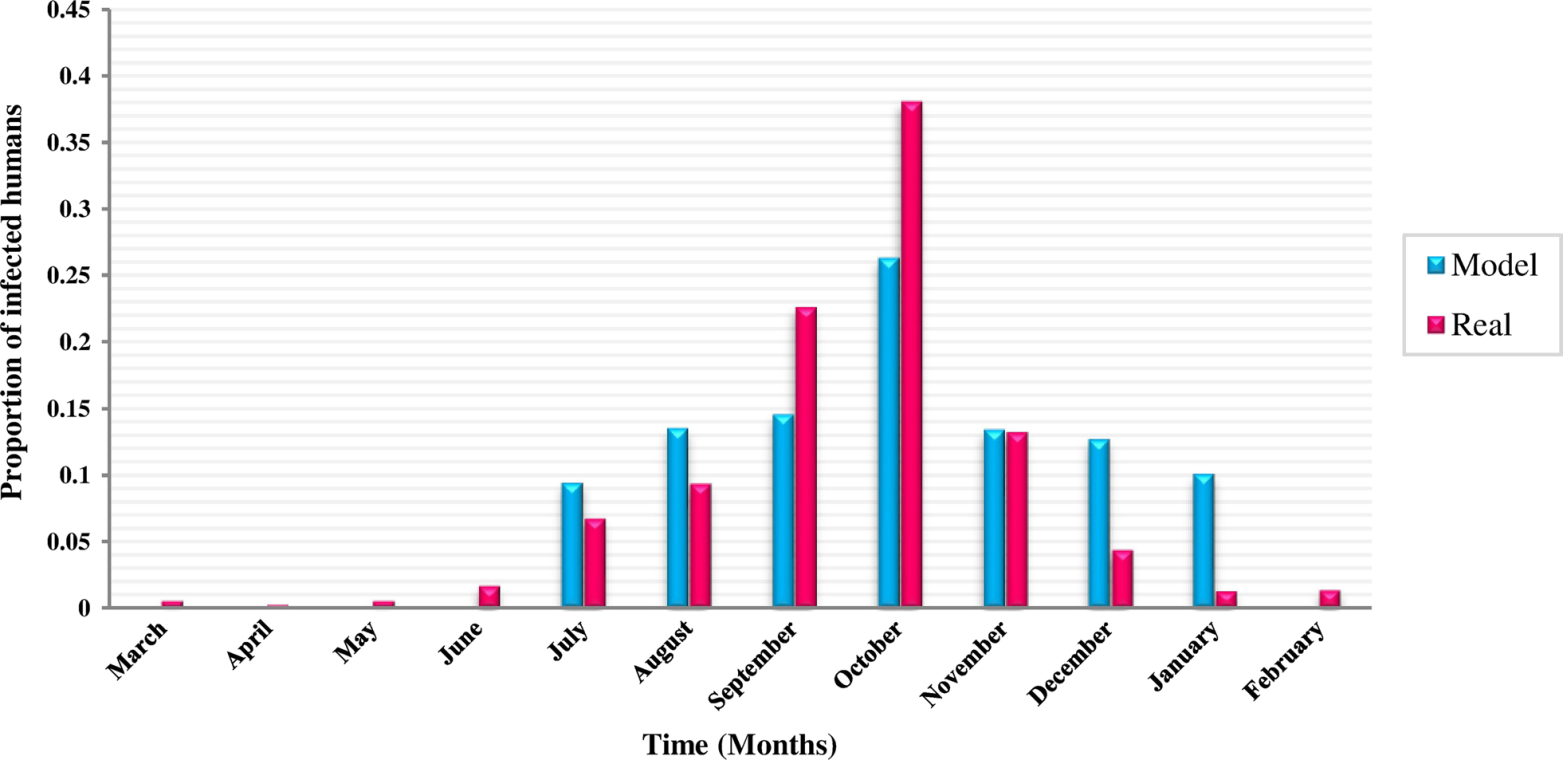
Comparison of simulation results with reality in terms of the temporal pattern of ZCL

To validate the temporal pattern of ZCL, we compare the proportion of infection per month obtained from the model with the reported ZCL data, as shown in Fig. 12. The blue bars show that the proportion of the infection started to rise in July, peaked in October, and then declined until the end of January. The ZCL data are reproduced quite well by the model. Therefore, the model provides a close representation of the ZCL epidemic.

## Discussion

Leishmaniasis is an endemic disease in several countries, which has also imposed social and economic damages. In each epidemiological study, three effective components are location, human, and time [50]. Many epidemiological aspects of diseases have been considered in several provinces of Iran, including Ilam [51], Kashan [52], Kerman [53] and northern Khorasan [54]. However, the above studies often ignored the environmental factors of leishmaniasis and only studied the characteristics of the individual patients and vector or reservoirs of their study area. Here, we developed a spatially explicit ABM integrated with GIS to explore the spatio-temporal spread of ZCL within an endemic region in northeastern Iran. We modeled the ZCL spread by explicitly representing the interactions between vectors, reservoir hosts, humans, and the environment and the spread of the epidemic using the SEIR epidemic model to understand the disease spread realistically.

We verified the model by using four scenarios. In scenario one, the results indicated that increasing the population size of each mobile agent leads to the growth of the infection. Accordingly, ZCL control programmes are based on reducing the population of rodents and sand flies [41]. Rajabi et al. [38] corroborated similar results. Other studies conducted in Golestan province indicated that in the areas where the number of sand flies and rodents has been higher, the number of ZCL cases has been higher, which is consistent with our results [8, 44, 55]. Moreover, this scenario showed that the spread of ZCL is similar to the traditional epidemiological curve.

In scenario two, we showed that a longer infectious period caused a greater infection during an epidemic. Since an increase in the infectious period, an infected human being can have more time to expose susceptible sand flies. It leads to developing disease quickly. Therefore, reducing the infectious period (i.e. applying fast treatment) can be a suitable control strategy to deal with the increased severity of the disease. Raising public awareness of ZCL and rapid diagnosis of the disease in areas with limited access to health facilities lead to reduce scarring and, in turn, early treatment. This scenario is compatible with the results of previous epidemiological studies on the disease spread [56, 57].

In the third scenario, by comparing Fig. 8a, b, it can be stated that an infected sand fly plays a more effective role in disease spread relative to an infected rodent. Therefore, to reduce the spread of ZCL, it might be most beneficial to focus on reducing the number of sand flies with the disease rather than reducing the rodent population in endemic areas. Currently, the ZCL control programs in Iran include the control of rodents and personal protection of sand flies bite. When the epidemic of the disease occurs, indoor residual spraying (IRS) does the control of sand flies. This method is much more effective than the rodent control policies and quickly reduces the incidence of ZCL in the endemic regions [41]. Therefore, the outcomes of this scenario correctly illustrate the accuracy of doing this control method in Iran.

In scenario four, we indicated how the restricting movement leads to reduce the severity of the epidemic. This finding is supported by relevant studies [37–39] and can be contributed to the fact that the most favorite habitats of rodents and sand flies are located outside of the villages in the case study. Thus, humans can do more activities and, as such, have a more chance to become infected as they face more sand flies in the infected areas. As a result, the disappearing of humans in the habitats of rodents and sand flies is a key factor for enhancing human immunity level against sand fly’s bites.

Moreover, we showed that finding evidence for the validity of the model by challenging them with ZCL outbreak data is possible. The results of our proposed model indicated that the outbreak of ZCL principally arises from the desert and low altitude areas and riverside population centers. Therefore, most disease cases occur in these regions. Studies in Golestan province showed that most of the ZCL cases occur in arid and semi-arid areas with lower altitudes, which confirm our results [8, 44].

Our results also showed that the spread of ZCL has a particular temporal pattern. In other words, most of the prevalent cases occur in the fall. This finding could be due to the latent period of the disease (four months) and inactivity of sand flies in fall and winter. As most of the bites occur in early summer, expected to that humans are infected in fall by spending this exposed period. Besides, the inactivity of sand flies in fall and winter explained the reducing infections in early summer. It could be due to unfavorable climatic conditions for the survival of sand flies in this period of a year. These results confirm the previous studies on the temporal pattern of ZCL in the study area [7, 44, 58–60]. Despite previous studies [61, 62] that did not consider the latent period of the disease for humans, we clarified the causes behind the particular temporal pattern of the disease by modeling this parameter (E_H_). We recommend that during the activity period of sand flies, particularly the beginning of the warm seasons, health authorities should use the control policies to reduce disease outbreak, such as minimizing the amount of exposed (uncovered) skin, applying insect repellent, staying in well-screened or air-conditioned areas and using insecticide spray.

We intend to extend this work in the future to model even more realistic situations, including the adaptive behaviors of humans, which can help reduce the spread of ZCL. Some specific adaptive behaviors that could be added to our model include changes in human behavior to avoid sand flies if they have encountered sand flies previously, sand fly and rodent population control, and healthcare-seeking behaviors if the infection is suspected. To represent human movements as more realistic behavior, a distance decay function could be applied in future works. In addition, future works should consider neglected influencing factors, which are effective on the ZCL epidemic, including climatic data (precipitation, humidity, temperature, etc.), culture, and the lifestyle of the population at risk to provide a clearer understanding of the ZCL spread. Besides, there is no connection between the age of each human and his or her probability of being infected by the ZCL since we attempted to keep the model as simple as possible. However, these impacts do happen in real conditions, and they should be considered in future studies.

## Conclusions

This survey creates a good match between the simulated and reported spatio-temporal trends, which demonstrates the remarkable power of data-intensive computing and its profound effects in the future, GIScience research. This work accentuates the role of ABM to explore the explicit representation of spatio-temporal patterns of ZCL and mobility of agents; especially, it supports the modeling of spatial heterogeneity. Although the model has been implemented in Maraveh Tappeh County, it can be easily extended to other endemic areas that provided relevant ZCL data and to other vector-borne diseases by adjusting a few landscapes and socioeconomic parameters. In this study, we developed an ABM integrated with an improved SEIR epidemic model to understand the spatiotemporal behavior of ZCL realistically. We investigated the model based on four scenarios. Through these scenarios, the model indicated how increasing the population could lead to a significant increase in new cases. The results confirmed that reducing the infectious period can be one of the effective measures to slow the transmission of the disease. The vector control policies can have a more significant impact to combat the disease than other measures. The results also showed that adopting restrictions on human movements lead to reduce the intensity of the epidemic. Besides, applying control policies at the beginning of the warm seasons could help health policymakers. Moreover, using the results of the spatial spread of the disease across time, health authorities can allocate the facilities to high-risk prone areas as properly.

## Supplementary information

**Additional file 1.** WinRAR archive (Model.rar) contains Source code.txt, GIS_Layers, Leishmania.nlogo (the model), and readme.txt (a guideline for running the model).

## Data Availability

The datasets used and, or analyzed during the present study are available from the corresponding author upon reasonable request.

## Abbreviations

ABM: Agent-based model
ACL: Anthroponotic cutaneous leishmaniasis
CDC: Center for Disease Control and Prevention
CL: Cutaneous leishmaniasis
DEM: Digital elevation model
GIS: Geospatial Information System
IRS: Indoor residual spraying
LUR: Land-use regression
NCC: National Cartographic Center
NDVI: Normalized differentiated vegetation index
SEIR: Susceptible-exposed-infected-recovered
UML: Unified modeling language
USGS: United States Geological Survey
VL: Visceral leishmaniasis
ZCL: Zoonotic cutaneous leishmaniasis
